# Early detection of tumour immune-rejection using magnetic resonance imaging

**DOI:** 10.1038/sj.bjc.6600814

**Published:** 2003-04-01

**Authors:** D-E Hu, D A Beauregard, M C Bearchell, L L Thomsen, K M Brindle

**Affiliations:** 1 Department of Biochemistry, University of Cambridge, 80 Tennis Court Road, Cambridge CB2 1GA, UK; 2 Immunomodulation Section, Immunotherapeutics Department, GlaxoSmithKline, Gunnels Wood Road, Stevenage SG1 2NY, UK

**Keywords:** tumours, immunotherapy, magnetic resonance imaging

## Abstract

Dynamic contrast agent-enhanced magnetic resonance imaging measurements of the perfusion of an immunogenic murine tumour showed that immune rejection was preceded by an increase in the apparent vascular volume of the tumour. This increase in vascularity, which has been observed previously in other tumours undergoing immune rejection, was confirmed by histological analysis of tumour sections obtained postmortem. Magnetic resonance imaging measurements similar to this could be used in the clinic to monitor the early responses of tumours to immunotherapy, before there is any change in tumour growth rate or volume.

The early detection of tumour responses to therapy will be valuable both in the development of new therapies and in their subsequent implementation in the clinic, where early response detection could be used to tailor therapy to individual patients.

The most widely used criterion for tumour response has been tumour shrinkage, however with some therapies, and particularly during early stage clinical trials, tumours may show a positive response to therapy in the absence of any change in tumour volume. For example, the antivascular drug combretastatin can produce dramatic changes in tumour blood flow without significantly affecting tumour volume (Chaplin *et al*, 1999). Magnetic resonance imaging (MRI) is widely used in the clinic for tumour detection and for monitoring tumour shrinkage post-therapy. Dynamic MRI measurements of contrast agent inflow into tumour vasculature and interstitial space, following intravenous injection of the agent, can both enhance the sensitivity of tumour detection as well as giving information on vascular volume and permeability (Tofts, 1997). Such measurements were used in the recently completed Phase I clinical trials of combretastatin to detect changes in tumour blood flow following drug treatment (Galbraith *et al*, 2000; Dowlati *et al*, 2002). These measurements are part of a growing family of so-called ‘molecular imaging’ methods in which specific molecules, which report on some aspect of tissue biochemistry or physiology, are detected through the changes that they produce in image contrast. In this case, the image was created by MR; however, molecular probes have also been developed for optical and radiological detection (Weissleder, 2001; Weis[43]sleder and Mahmood, 2001). The probes thus identify, amplify and translate information about underlying tissue biochemistry and physiology into the image. These techniques can be used in the laboratory, for basic science studies, and in the clinic, where they can provide specific information about responses to therapy. For example, in the case of MRI, contrast agents have been developed for imaging pH (Zhang *et al*, 1999; Aime *et al*, 2002), for monitoring gene expression (Louie *et al*, 2000; Weissleder *et al*, 2000), for detecting angiogenic tumour vasculature (Sipkins *et al*, 1998) and for detecting tumour cell apoptosis following chemotherapy (Zhao *et al*, 2001).

Tumour immunotherapy has, until recently, met with limited success. However, a better understanding of how cytotoxic T lymphocytes respond to tumour cells, the molecular signalling pathways that regulate responsiveness and the mechanisms by which tumours evade immune surveillance coupled with the identification of tumour antigens has led to renewed interest in this therapeutic approach (Rosenberg, 2001; Smyth *et al*, 2001; Pardoll, 2002). Indeed, there are many examples of immunotherapies that are currently in clinical trials (http://www.cancer.gov/clinical_trials).

We show here how dynamic contrast agent-enhanced MRI (DCE-MRI) measurements can be used to detect changes in the vascularity of tumours undergoing immune rejection, prior to a reduction in tumour volume. We suggest that similar measurements could be used clinically to detect the early response of tumours to immunotherapy.

## MATERIALS AND METHODS

### Antibodies and other reagents

Rabbit antiovalbumin antibody was purchased from Research Diagnostics Inc., NJ, USA. Rabbit polyclonal antibodies recognising CD4, CD8-*α*, goat polyclonal antibodies recognising CD-68 (M-20) and a secondary anti-rabbit IgG staining system (‘Immuno-Cruz staining system’) were purchased from Santa Cruz Biotechnology Inc., USA. Rabbit anti-human Factor VIII antibody was purchased from DAKO, Copenhagen, Denmark. Fluorescein isothiocyanate (FITC)-conjugated monoclonal antibodies recognising rabbit and goat IgGs were purchased from Sigma (Gillingham, Dorset, UK). All other reagents were of analytical grade.

### Cell lines and tumour implantation

The E.G7-OVA cell line was originally derived from the murine thymoma line, EL-4, by transfection with a neomycin-selectable vector expressing full-length chicken ovalbumin (Moore *et al*, 1988). E.G7-OVA and EL-4 cells used in this study were taken from frozen stocks held at GlaxoSmithKline, Stevenage, UK and were originally obtained from Francis Carbone (Scripps Clinic, La Jolla, CA). They were cultured as a suspension in RPMI 1640 medium (Invitrogen Ltd, Paisley, UK) containing 10% heat-inactivated foetal calf serum (PAA Laboratories, GmbH Linz, Austria), 2 mM L-glutamine, penicillin (100 units ml^−1^) and streptomycin (100 *μ*g ml^−1^). Selection of E.G7-OVA cells was maintained using culture medium containing 400 *μ*g ml^−1^ G418.

Female C57BL/6 mice were purchased at 6–8 weeks of age from Charles River Ltd (UK). Tumour cells (5 × 10^6^) were injected subcutaneously into the shaved flanks of mice. Tumour size was measured using calipers and is reported as the product of the two largest perpendicular diameters (mm^2^). All experiments were conducted in compliance with a project licence issued under the Animals (Scientific Procedures) Act 1986 and were designed with reference to the UKCCCR guidelines for the welfare of animals in experimental neoplasia. The work was approved by a local ethical review committee.

### Tumour histology and immunohistochemical examination

Tumours were fixed in 10% formalin and embedded in paraffin. Five-micrometre thick sections were cut and stained with haematoxylin and eosin (H&E) or Masson's trichrome stain (Sigma). For immunoperoxidase staining, the rabbit primary antibodies were detected using a biotinylated anti-rabbit IgG secondary antibody and horseradish peroxidase (HRP)-conjugated to streptavidin using the Immuno-Cruz staining system. The formalin-fixed and paraffin-embedded sections were deparaffinised and then heated to 95°C for 10 min in 10 mM sodium citrate buffer, pH 6.0. They were then incubated with a peroxidase- and then a serum-block and incubated with an appropriately diluted primary antibody before incubation for 30 min with the biotinylated secondary antibody and 30 min with the HRP–streptavidin complex. The target protein was visualised by incubation for 30 min with a peroxidase substrate, using diaminobenzidine tetrahydrochloride (DAB) as the chromogen. As a negative control, the sections were treated with nonimmune rabbit serum. For immunofluorescence staining, the slides were prepared as described for immunoperoxidase staining, except that the peroxidase-block step was omitted. Slides were incubated with an appropriately diluted primary antibody before detection with a fluorescein-conjugated secondary antibody.

Flow cytometry was also performed on cell suspensions prepared from tumour tissue and on the tumour cells grown in culture. The cells were incubated with the appropriate primary antibody and then with a secondary fluorochrome-conjugated antibody. Flow cytometry was performed using a FACScan Instrument and analysed with Lysys II software (Becton Dickinson, Mountain View, CA, USA). Tumour cell suspensions were prepared from tumour tissue by grinding the excised tumour through a stainless-steel mesh (Dyall *et al*, 1999) into serum-free RPMI 1640 medium.

Vascular volumes were analysed using an axial strip sampling technique (Mayhew and Sharma, 1984). Sections, stained with Masson's trichrome stain or with antibody to Factor VIII, were examined at × 200 magnification and the images relayed to a computer. The volume fraction occupied by the blood vessels was estimated using a 486-point square lattice (18 × 27) with a field-of-view of 0.209 mm^2^. Functional vessels were determined in a similar manner following intravenous injection of carmine red dye (Sigma) (Kimura *et al*, 1986). In these experiments, 1 ml of a saline solution containing 10% (w v^−1^) carmine red dye and 5% (w v^−1^) gelatin were injected into the tail veins of anaesthetised animals. The mice died within 30 s of injection and were then cooled below 4°C for 1 h in order to solidify the gelatin in the blood vessels. The tumours were excised and fixed in a solution of 10% formalin in saline. Sections (5 *μ*m thick) from the fixed and paraffin-embedded tumours were then cut, deparaffinised and counterstained in 2% light green dye (Sigma).

### Determination of vascular endothelial growth factor levels in serum, tumour cells and whole tumours

Vascular endothelial growth factor (VEGF) was detected by ELISA, using an antibody raised against recombinant mouse VEGF and a kit purchased from R&D Systems, Inc., Minneapolis, USA. Information provided by the manufacturer indicates that this antibody can be used to determine the relative mass values for natural mouse VEGF. Serum was obtained from blood samples taken by intracardiac puncture. The blood was allowed to clot for 30 min before centrifugation for 10 min at 1000 **g**. The serum was removed and either assayed immediately for VEGF or stored at −85°C until assayed. Vascular endothelial growth factor was measured in tumour cells grown in culture by first harvesting cells from a growing culture, washing them in ice-cold PBS and then resuspending the cell pellet, containing approximately 2 × 10^8^ cells, in 25 ml of fresh extraction buffer in a tight-fitting Potter homogeniser. The buffer contained 50 mM Tris-HCl, pH 8.2, 2 mM dithiothreitol, 2 mM EDTA and 1% Triton X-100. After 10 strokes with the pestle, the resulting cell extracts were kept on ice for 30 min and then centrifuged for 15 min at 2000 **g**, to remove any insoluble material. The supernatants were removed and either assayed immediately for VEGF or stored at −85°C until assay. Vascular endothelial growth factor levels in tumours were measured by first preparing tumour homogenates. After weighing, excised tumours were placed in 10 ml of ice-cold extraction buffer in a Potter homogeniser and homogenised using 25 strokes of the pestle. The resulting homogenates were kept on ice for 30 min and then centrifuged for 15 min at 2000 **g**. The supernatants were removed, passed through a 0.45 *μ*m Millipore filter and either assayed immediately for VEGF or stored at −85°C until assay. Protein concentrations in the serum, cell and tumour extracts were determined by the method of Bradford (1976), using a Bio-Rad Protein Assay kit (Bio-Rad Laboratories, CA, USA).

### Magnetic resonance imaging

Experiments were performed in a 9.4-T vertical bore (8.9 cm diameter) superconducting magnet (Oxford Instruments, Oxford, UK), interfaced with a Varian Associates UnityPlus spectrometer and Sun workstation running Vnmr 5.3B software. An unshielded gradient set (Varian Associates) was used with a home-built probe incorporating a two-turn surface coil probe (20 mm diameter) tunable to frequencies of 400 MHz (^1^H-imaging) and 162 MHz (^31^P-spectroscopy), although only proton imaging was performed in this study. Animals were anaesthetised by intraperitoneal (i.p.) injection of Hypnorm/Hypnovel/dextrose–saline in the ratio 5 : 4 : 31 (10 ml kg^−1^ body weight) and immobilised in a cradle that held the tumour in the centre of the surface coil. Hypnorm was from Jansen Pharmaceuticals (High Wycombe, UK) and Hypnovel was from Roche (Welwyn, UK). The dextrose–saline solution contained 4% dextrose and 0.18% saline. Dextrose–saline (0.5 ml) was also given i.p. to reduce animal dehydration. Anaesthesia was maintained, for up to 5 h, with 1.75-h i.p. injections of Hypnorm–dextrose–saline solution in the ratio 1 : 19 (5 ml kg^−1^ body weight). A flow of warm air was used to maintain body temperature. Tumour perfusion was measured by using a series of rapidly acquired T_1_-weighted spin echo images (TE=12.3 ms, TR=130 ms) to monitor the inflow and subsequent efflux from the tumour of the MRI contrast agent, gadolinium diethylenetriaminepentaacetate (Gd-DTPA) (Magnevist, Schering), as described previously (Beauregard *et al*, 1998,^2001^). Gd-DTPA, diluted to 40 mM with sterile saline (0.9% sodium chloride), was administered intravenously (i.v.) through a tail vein catheter over a period of ∼30 s to give 200 *μ*mol kg^−1^ body weight. Images were acquired immediately before and for up to 30 min after contrast agent injection from a 1 mm-thick slice and a field-of-view of 20 times; 20 mm^2^ over a 256 × 128 (phase encode) data matrix, that was zero-filled to 256 × 256 data points. For calculation of the paramagnetic contribution to the relaxation rate (*R*_1p_), an image with TR=3 s was acquired at 30 min after contrast agent injection. Image pixel intensities in the short TR experiments were converted to *R*_1p_ values using the expressions derived by Kennedy *et al* (1994). *R*_1p_ is directly proportional to the concentration of the contrast agent and can thus be used to assess quantitatively the perfusion of a tumour by the contrast agent. These *R*_1p_ maps were transformed into maps of Gd-DTPA concentration, using the relaxivity of Gd-DTPA at this field strength (3.88 mM^−1^ s^−1^) (Thelwall *et al*, 2001). The procedure detailed by Su *et al* (1994), which enables separation of the vascular and extravascular components, was then applied and the vascular volumes of the tumours determined. In doing this, the data of Furman-Haran *et al* (1996) were used to derive the arterial input function, where a biexponential function characterises the plasma concentration of Gd-DTPA in mice after i.v. injection. Perfusion can also be assessed in a semiquantitative manner by measuring signal enhancement in the presence of the contrast agent. The enhancement of signal intensity in the tumours following contrast agent injection was calculated, on a pixel-by-pixel basis, by subtracting pixel intensities in the precontrast images from the intensities of the corresponding pixels in the postcontrast images and then dividing by the pixel intensities in the precontrast images.

A further estimate of vascular volume was made using the macromolecular contrast agent, BSA–Gd-DTPA, in which bovine serum albumin (BSA) is conjugated to Gd-DTPA. The agent was prepared as described by Ogan *et al* (1987) and injected i.v. to give a concentration of 250 mg kg^−1^ body weight. The imaging protocol was the same as that used for the Gd-DTPA experiment except that a long-TR image was not acquired and therefore only signal enhancement after contrast agent injection was calculated.

## RESULTS

### Tumour model

**Figure 1:**
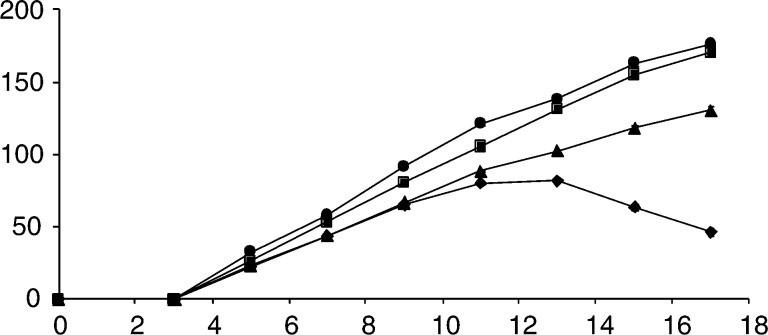
Growth rates of implanted tumours following subcutaneous injection of 5 × 10^6^ cells at *t*=0 days. (•) EL-4 tumours (*n*=30); (▪) E.G7-OVA tumours, arising from cells implanted at nominal passage numbers 13 – 20 (*n*=28); (▴) E.G7-OVA tumours arising from cells implanted at nominal passage numbers 7 – 12 (*n*=26); (♦) regressive E.G7-OVA tumours (18 arising from cells implanted at nominal passage numbers 7 – 12 and five arising from cells implanted at nominal passage numbers 13 – 20). The symbols represent the mean±s.e.m. of the volumes reported as the product of the two largest perpendicular diameters. Where they are not visible, the error bars lie within the symbols.

The E.G7-OVA thymoma, a derivative of the H-2^b^ thymoma EL-4, which has been transfected with a vector expressing a full-length chicken ovalbumin cDNA (Moore *et al*, 1988) and displays the immunodominant epitope of chicken ovalbumin on its surface (Shastri and Gonzalez, 1993), has been a relatively widely used model in studies of immune responses to tumour cells (Zhou *et al*, 1992; Minev *et al*, 1994; Brossart *et al*, 1997). The doubling times for the EL-4 and E.G7-OVA tumour cells *in vitro* were 11.0±0.1 (s.e.m., *n*=3) and 11.99±0.4 (s.e.m., *n*=3) h, respectively. The small difference in these doubling times is not statistically significant. Following subcutaneous injection of 5 × 10^6^ EL-4 or E.G7-OVA cells, C57BL/6 mice developed palpable tumours within 5 days (Figure 1

). There were no significant differences in the growth rate of EL-4 tumours (*n*=30) and of those E.G7.OVA tumours that arose from cells that had been implanted between nominal passage numbers 13–20 (*n*=28). However, E.G7-OVA tumours that arose from cells that had been implanted between nominal passage numbers 7–12 grew more slowly than EL-4 tumours and those E.G7-OVA tumours that arose from cells implanted between the later nominal passage numbers 13–20 (*P*<0.001, ANOVA). Twenty-three out of 73 (32%) E.G7-OVA tumours underwent spontaneous regression, including 18 out of 42 (43%) that developed from cells implanted between nominal passage numbers 7–12 and five out of 26 (19%) that developed from cells implanted between nominal passage numbers 13–20. This difference in the tumorigenicity of early and late passage cells was judged to be significant by a *ψ*^2^ test (*ψ*^2^=5.9, *P*<0.025).

Flow cytometry showed that an earlier passage of the E.G7-OVA cell line (nominal passage number 10) expressed significantly (*P*<0.01) higher levels of ovalbumin (842±95, mean±s.e., *n*=3) when compared to a later passage (402±51, mean±s.e., *n*=3) (nominal passage number 13). The EL-4 cell line showed no significant detectable signal (3.0±0.1, mean±s.e., *n*=3). The numbers represent fluorescence intensity in arbitrary units. Immunohistochemistry showed significant staining for ovalbumin in sections of E.G7-OVA tumours, but no detectable staining in sections of EL-4 tumours (data not shown). Flow cytometric analysis of ovalbumin expression on cells prepared from disaggregated tumours showed that ovalbumin expression was significantly higher in regressing E.G7-OVA tumours than in those that continued to grow (Table 1

**Table 1:** Flow cytometric analysis of ovalbumin expression and immune cell infiltration in EL-4, progressive E.G7-OVA and regressive E.G7-OVA tumours

	**Tumour type**		
**Antigen**	**EL-4**	**E.G7-OVA (regressive)**	**E.G7-OVA (progressive)**
Ovalbumin	3.4±0.2	1608±480^a^	309±66
CD8	150±23	272±27^a^	174±19
CD68	64±17	177±35^b^	99±29

At 13 days after implantation, single-cell suspensions were prepared by disaggregating solid tumour material. These cell suspensions were then incubated with the relevant primary antibody and fluorochrome-conjugated secondary antibody, as described in the Materials and Methods section. Three tumours were used in each group and the numbers represent the mean±s.e.m. of the fluorescence intensities in arbitrary units.

a Statistically significantly different from EL-4 and progressive E.G7-OVA tumours, *P*<0.01;

b statistically significantly different from EL-4 tumours, *P*<0.01.

).

### Tumour histology

Previous work has shown that CD8^+^ T cells are responsible for rejection of E.G7-OVA tumours and that administration of an anti-CD4 monoclonal antibody, that mediates regression of established E.G7-OVA tumours, leads to increased infiltration of CD8^+^ cells (Vasovic *et al*, 1997; Dyall *et al*, 1999). Flow cytometric analysis of disaggregated tumours showed that there was increased numbers of CD8^+^ and CD68^+^ cells in regressing E.G7-OVA tumours when compared to EL-4 tumours and progressive E.G7-OVA tumours (Table 1). CD68 is antigen expressed on murine macrophages (Ramprasad *et al*, 1995). The increased infiltration of CD8^+^ cells and macrophages was confirmed by immunohistochemistry (data not shown).

The levels of VEGF in the serum of EL-4 and E.G7-OVA tumour-bearing mice, in EL-4 and E.G7-OVA tumour homogenates and in extracts of EL-4 and E.G7-OVA tumour cells grown *in vitro* were measured by ELISA (see Materials and Methods section). Vascular endothelial growth factor was significantly higher (*P*<0.01) in the serum of tumour-bearing mice (18.6±1.0 pg ml^−1^, E.G7-OVA; 18.4±1.3 pg ml^−1^, EL-4) than in controls (9.2±1.1 pg ml^−1^). There were two mice in each group and four measurements of VEGF per group. There were also significantly higher (*P*<0.01) levels of VEGF in E.G7-OVA tumour homogenates (16.9±0.4 pg mg^−1^ protein) than in EL-4 tumour homogenates (12.5±0.5 pg mg^−1^ protein) (two mice in each group and four measurements per group). The E.G7-OVA tumours arose from cells implanted at nominal passage 12 and were taken for extraction 13 days after tumour cell implantation. There was no significant difference in the levels of VEGF in extracts of E.G7-OVA and EL-4 cells, 4.8±0.6 and 5.3±0.2 pg *μ*g^−1^ protein, respectively.

**Figure 2:**
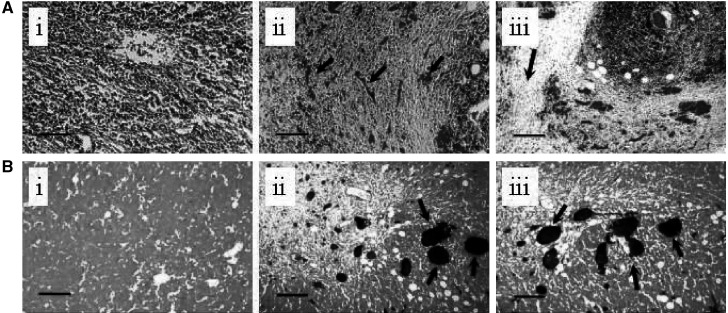
Histological features of spontaneous regression in E.G7-OVA tumours. Tumour sections (5 *μ*m thick) were stained with Masson's trichrome (**A**) or carmine red dye counterstained with light green (**B**). In **A** (ii and iii) the tumour cells can be seen to be surrounded by fibroblasts and capillaries filled with red blood cells (arrowed). There is also evidence of collagen deposition, which stains green with this dye (arrowed in **A** iii). **A**(i) is a representative section from an EL-4 tumour. The tumour sections shown in **B** (ii and iii) were obtained following injection of the animal with carmine dye. Functional vessels are stained red in these sections (arrowed). **B**(i) is a representative section from an EL-4 tumour. The bars are 400 *μ*m long.

In addition to the lymphocytic infiltrate observed in regressing E.G7-OVA tumours, there was also clear evidence of an angiofibroblastic response (Bodurtha *et al*, 1976; Lynch *et al*, 1978; Martin *et al*, 1987; Shaw *et al*, 1989; Vaage, 1992), with proliferation of fibroblasts and capillary vessels and encapsulation of the tumour in a fibrous, collagen containing, capsule (see Figure 2

). Fibrous tissue and stroma stain bluish-green, and blood vessels containing red cells stain red. The functional vascular volume, as assessed from carmine dye injection (see Materials and Methods section), was significantly increased in regressing E.G7-OVA tumours as compared to progressive E.G7-OVA tumours and EL-4 tumours (see Table 2

**Table 2:** Vascular volumes in EL-4, progressive E.G7-OVA and regressive E.G7-OVA tumours

	**Tumour type**		
**Stain**	**EL-4**	**E.G7-OVA (regressive)**	**E.G7-OVA (progressive)**
Masson's trichrome	0.9±1.5	8.7±5.5^a^	1.1±1.6
Carmine red	0.7±1.2	8.9±5.3^a^	1.1±1.7
Factor VIII	0.7±1.4	6.1±4.0^a^	0.8±1.2
DCE MRI (Gd-DTPA)	2.1±1.2	8.9±2.7^a^	4.4±1.4^b^

Tumour sections stained with either Masson's trichrome or an antibody against Factor VIII, or obtained from mice that had been injected with carmine red dye, were observed at × 200 magnification and the resulting images relayed to a computer screen. Vascular volume, as a percentage of tumour volume, was estimated by an axial strip sampling technique, as described in the Materials and Methods section. The data are the mean percentage±s.d. for 90 tumour areas stained with Masson's trichrome, 30 areas stained with antibody against Factor VIII and 90 areas from carmine dye-injected tumours. Three tumours were used in each group. Also given are vascular volumes (as mean percentage±s.d.) determined using dynamic contrast agent-enhanced MRI, as described in the Materials and Methods section. Respectively, three, five and five tumours were used.

a Statistically significantly different from EL-4 and progressive E.G7-OVA tumours, *P*<0.01.

b Statistically significantly different from progressive E.G7-OVA tumours, *P*<0.05.

). The dye was injected and the tumours excised between days 13 and 15, at which times the regressing E.G7-OVA tumours could be identified by cessation of tumour growth or even by a small reduction in size (see Figure 1). The increased vascular volume was confirmed in sections stained with an antibody that recognises Factor VIII on vascular endothelial cells and in sections stained with Masson's trichrome (Table 2).

### Magnetic resonance imaging

**Figure 3:**
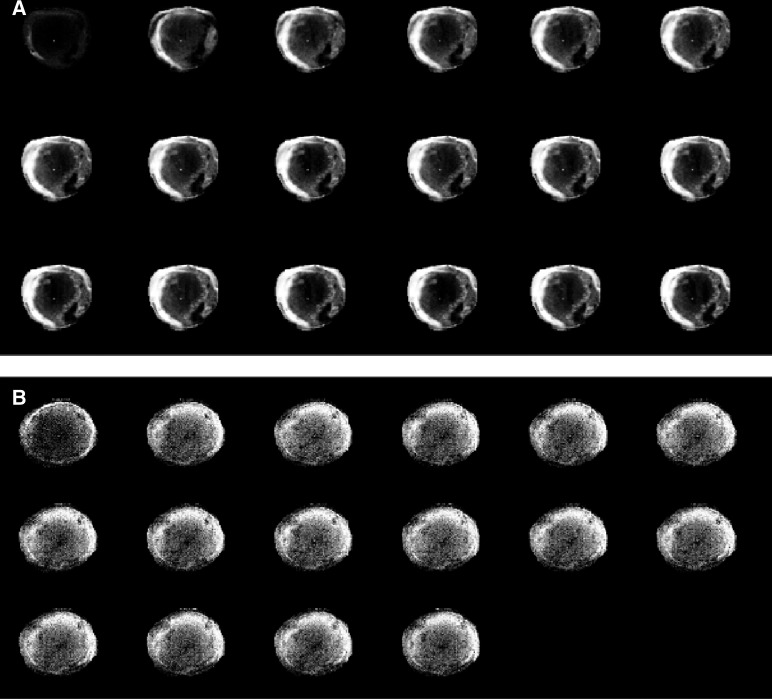
A series of *T*_1_-weighted MR images acquired from a regressive E.G7-OVA tumour (**A**) and an EL-4 tumour (**B**) following i.v. injection of the MRI contrast agent, Gd-DTPA. The first images in the series were acquired prior to contrast agent injection. The subsequent images (reading from left-to-right and top-to-bottom) were acquired at 2 min intervals. The presence of the contrast agent increases signal intensity in the images and these increases are proportional to its concentration.

Perfusion of EL-4 and E.G7-OVA tumours was assessed by monitoring the inflow of an MRI contrast agent, Gd-DTPA, following intravenous injection between days 10 and 12 after tumour implantation. The inflow of the agent was measured using a series of rapidly acquired, *T*_1_-weighted, spin echo images, in which the increase in signal intensity was proportional to the concentration of the contrast agent. Typical image series for an EL-4 tumour and an E.G7-OVA tumour, which went on to regress, are shown in Figure 3

**Figure 4:**
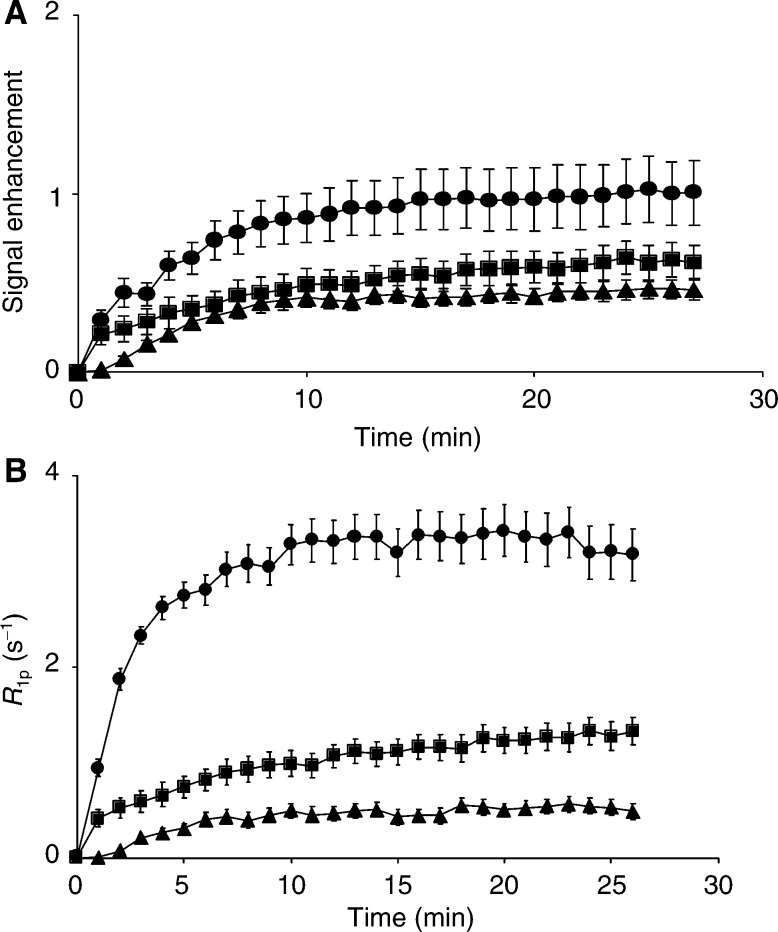
Time-dependent changes in signal enhancement (**A**) and *R*_1p_ values (**B**) in dynamic contrast agent-enhanced *T*_1_-weighted images obtained from EL-4 (▴), progressive E.G7-OVA (▪) and regressive E.G7-OVA (•) tumours. *R*_1p_, the paramagnetic contribution to the relaxation rate, is equivalent to the contrast agent concentration. The symbols represent the mean±s.e.m. (*n*=5). The numbers for signal enhancement were obtained from the entire tumour cross-section. However, because of difficulties in estimating *R*_1p_ values in the relatively poorly perfused regions in the centres of some tumours, these were calculated for a 20 pixel-wide band in the tumour peripheries, where vessel density was higher.

. The increased signal intensity in the regressive E.G7-OVA tumour (Figure 3A) shows clear evidence of vascular proliferation in the tumour periphery. Histological analysis also showed that the vascular proliferation accompanying immune rejection occurred predominantly at the tumour edge (data not shown). These MR images show, in a qualitative way, that tumour perfusion was increased in the E.G7-OVA tumour that went on to regress, when compared to the nonregressing EL-4 tumour. This was confirmed, in a more quantitative way, by plotting mean signal enhancement across the whole tumour *vs* time, for 5 EL-4, 5 E.G7-OVA progressive and 5 E.G7-OVA regressive tumours (Figure 4

). Signal enhancement in the regressive E.G7-OVA tumours was significantly greater (*P*<0.0001, one way ANOVA) than in either the EL-4 or progressive E.G7-OVA tumours. These data can be used to estimate perfusion quantitatively by converting the increase in image pixel intensities, in the presence of the agent, into *R*_1p_ values, as described previously (Kennedy *et al*, 1994; Beauregard *et al*, 1998,^2001^) and in Materials and Methods section. *R*_1p_ is the paramagnetic contribution to the longitudinal relaxation rate and is directly proportional to the concentration of the contrast agent. The centres of these tumours were relatively poorly perfused and therefore estimates of *R*_1p_ values in these regions had a large error associated with them, as we have observed previously in other tumours (Beauregard *et al*, 2001,^2002^). Therefore, perfusion was assessed by measuring the mean increase in *R*_1p_ values in a 20 pixel-wide band in the tumour periphery. Plots of the mean increase in *R*_1p_ values, following intravenous injection of Gd-DTPA, in EL-4, progressive E.G7-OVA and regressive E.G7-OVA tumours, are shown in Figure 4. These show that there was a significant (*P*<0.001, *n*=5, ANOVA) increase in the perfusion of those E.G7-OVA tumours that went on to regress, when compared to EL-4 and progressive E.G7-OVA tumours. These data were also analysed to estimate tumour vascular volumes, using the model described by Su *et al* (1994). The pharmacokinetic data of Furman-Haran *et al* (1996) for Gd-DTPA in mouse plasma were used to derive an arterial input function. The vascular volumes of regressive E.G7-OVA tumours were significantly higher than those of both EL-4 (*P*<0.01) and progressive E,G7-OVA tumours (*P*<0.01, Student's *t*-test). The vascular volumes of the progressive E.G7-OVA tumours were also significantly higher than those of EL-4 tumours (*P*<0.05). The vascular volumes determined by MRI were similar to those obtained using the carmine dye injection technique for the regressive EG7-OVA tumours, but significantly higher than those obtained using the dye in the EL-4 and progressive E.G7-OVA tumours (see Table 2). This may reflect the fact that the MRI estimate was taken from the tumour periphery, where vessel density is higher.

**Figure 5:**
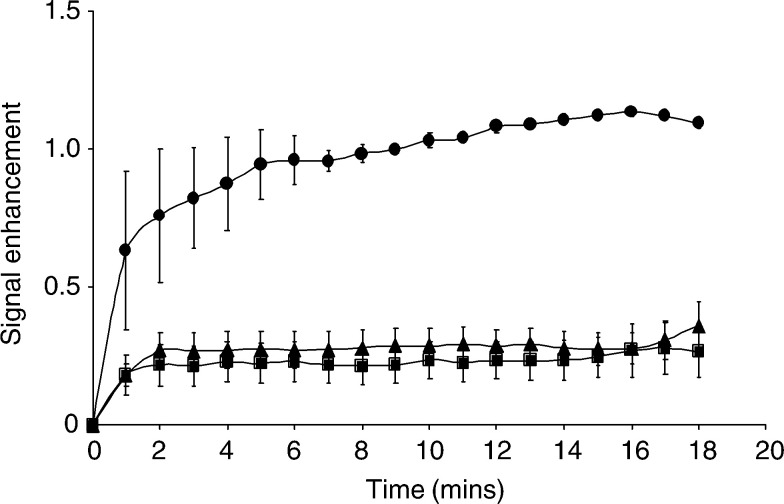
Time-dependent changes in signal enhancement in tumours using dynamic contrast-enhanced MRI with the macromolecular contrast BSA–Gd-DTPA. A series of *T*_1_-weighted spin echo images were acquired from EL-4 (▴, *n*=7), progressive E.G7-OVA (▪, *n*=6) and regressive E.G7-OVA (•, *n*=2) tumours. The symbols and error bars show the mean±s.e.m. The values were calculated for a 20 pixel-wide band in the tumour peripheries, where vessel density was higher. The high molecular weight agent is initially confined to the vasculature, so the greater signal enhancement of regressive E.G7-OVA tumours is indicative of a higher functional vascular volume fraction.

The high molecular weight contrast agent, BSA–Gd-DTPA, was also used to determine relative vascular volumes for the three tumour models. This contrast agent is initially contained in the plasma compartment, so allowing an estimate of vascular volume. In areas of permeable vasculature it then escapes into the interstitial space. The signal enhancement for the three tumours (Figure 5

) indicates that the vascular volume of regressive E.G7-OVA was greater than that of the other two tumour models, in agreement with the quantitative estimates obtained using Gd-DTPA and from histology. The increase in signal enhancement with time observed for regressive E.G7-OVA tumours (Figure 5) indicates a more permeable vasculature than in the EL-4 or progressive E.G7-OVA tumours.

## DISCUSSION

The clinical development of cytotoxic antitumour drugs has traditionally viewed tumour shrinkage as an objective indicator of drug efficacy. However, early Phase I/II clinical trials of antiangiogenic drugs have shown that they are mostly cytostatic, slowing or stopping tumour growth, without producing tumour regression. This has prompted the recommendation that in early stages of antiangiogenic drug development, we should seek to establish surrogate markers of drug efficacy (Cristofanilli *et al*, 2002). Since cytostasis is a reasonable outcome, in the first instance, and since we might reasonably expect to observe this during the development of other therapeutics, then perhaps this recommendation should be widened to include other therapeutic approaches. We have shown here how clinically applicable, noninvasive DCE MRI measurements of tumour perfusion can be used to detect the relatively early signs of tumour immune rejection, before there is a reduction in tumour growth rate or volume.

The E.G7-OVA tumour model has been widely used in studies of tumour immunotherapy procedures (Zhou *et al*, 1992; Minev *et al*, 1994; Brossart *et al*, 1997). The cell line, which has been transfected with a vector expressing full-length chicken ovalbumin cDNA, expresses on its surface an eight-residue peptide of ovalbumin in association with the MHC class I molecule H-2K^b^. Adoptive transfer experiments have demonstrated that OVA-specific CD8^+^ T cells can specifically protect animals against the growth of E.G7-OVA tumours, but that they cannot eradicate established tumours. Tumour eradication can be obtained, however, by injection of an antibody against CD4, which is expressed on E.G7-OVA cells *in vivo* (Vasovic *et al*, 1997). The antibody-mediated tumour rejection was shown to have an absolute requirement for CD8^+^ T cells and CD11b^+^ (phagocytic) cells. Rejection was accompanied by significant infiltration of CD8^+^ T cells, which was approximately five times greater than in control tumours (Dyall *et al*, 1999).

In the E.G7-OVA tumour model used in this study, we noted a significant level of spontaneous regression, which appeared to be correlated with the level of ovalbumin expression determined by flow cytometry. Tumours arising from early passage cells, which showed the highest levels of ovalbumin, underwent spontaneous regression with a significantly higher frequency (*P*<0.025) than tumours arising from later passage cells, which showed lower levels of ovalbumin (Figure 1). Tumour regression was accompanied by increased infiltration of CD8^+^ T cells, which was nearly double that in EL-4 tumours, and of macrophages (CD68^+^) (Table 1). What the antiovalbumin antibody is recognising on the surface of these E.G7-OVA cells and the factors responsible for the decrease in expression of the recognised epitope with cell passage number are not clear. Nevertheless, this tumour provided us with a model system in which we could evaluate MRI methods for detecting early signs of tumour rejection and thus methods for evaluating the early responses of tumours to immunotherapy.

Measurements of tumour perfusion showed that this was significantly increased in those E.G7-OVA tumours that went onto regress when compared to the EL-4 parent tumour and progressive E.G7-OVA tumours. Tumour perfusion was assessed by monitoring the inflow of an intravenously injected MRI contrast agent, Gd-DTPA, using a series of rapidly acquired *T*_1_-weigthed spin echo images (Figure 3). The presence of the contrast agent leads to signal enhancement in these images. The kinetics of signal enhancement were analysed using a pharmacokinetic model to obtain a vascular volume, as described by Su *et al* (1994). The initial increase in signal intensity, after contrast agent injection, was attributed in this model to vascular distribution. However, Gd-DTPA is a relatively small molecule and can leak rapidly out of the vasculature into the tumour interstitial space. Therefore, the vascular volume estimated using this model is an apparent volume, which includes the true vascular volume and a fast leakage volume. Nevertheless, there was a reasonable agreement between the vascular volume determined in this way for the regressive E.G7-OVA tumours and that determined by conventional histological methods in tumour sections obtained post mortem. The latter included determination of functional vascular volume using carmine dye injection, detection of intravascular red cells using Masson's trichrome stain, and staining of vascular endothelial cells using antibody against Factor VIII. The higher vascular volume determined by MRI in the EL-4 and progressive E.G7-OVA tumours may be a reflection of the fact that the MRI measurements were taken from the tumour periphery, where vessel density was higher.

The vascular proliferation observed here in regressing E.G7-OVA tumours has been observed previously during the spontaneous regression of melanoma (Bodurtha *et al*, 1976; Lynch *et al*, 1978; Shaw *et al*, 1989; Blessing and McLaren, 1992) and in other tumours (Junker *et al*, 1997). In melanoma, Shaw *et al* (1989) defined three stages of regression; early, in which there was lymphocytic infiltration and ‘degenerating’ tumour cells; intermediate, in which there were areas of proliferating fibroblasts associated with new blood vessels, and late, in which there was an absence of tumour cells and extensive fibrosis. It seems clear that, on this histopathological scale, our MRI measurements are detecting intermediate stages of immune rejection. The histological measurements, which were made on tumours excised 2–3 days after the MRI measurements, when evidence of tumour regression was apparent from cessation of tumour growth or a reduction in size, also showed evidence of later stages of regression. This included extensive fibrosis and also the presence of a collagen capsule surrounding tumour cells. The capsule appears to be laid down by infiltrating macrophages (Vaage and Harlos, 1991). The vascular proliferation may have been stimulated by angiogenic growth factors secreted by the infiltrating macrophages (Leek *et al*, 1996). Extracts prepared from tumours arising from early passage cells and excised 13 days after implantation, when there was evidence that they had ceased growth and were thus classified as regressive tumours, showed significantly higher levels of VEGF when compared to extracts of EL-4 tumours. Vascular endothelial growth factor is known to increase vascular permeability, and therefore the elevated levels in regressive E.G7-OVA tumours might explain the increased vessel permeability observed in the MRI experiments in which BSA–Gd-DTPA was used as the contrast agent (Figure 5). The serum of tumour-bearing mice also contained significantly higher levels of VEGF than serum from nontumour-bearing control animals, as has been observed previously in tumour-bearing animal and human subjects (Kondo *et al*, 1994).

The observation that tumour regression is preceded by an increase in vascular volume is at variance with an MRI study of allogeneic tumours in rat, where a decrease in tumour volume was shown to be preceded by a reduction in vascular volume (Su *et al*, 2000). However, there was no accompanying histology in the rat study and it is difficult to know at what stage of regression these tumours were at when studied by MRI. The differences between these studies might be resolved by extending, to earlier time points, measurements of contrast agent uptake in the E.G7-OVA tumours. However, this will be difficult because of their relatively small size at these earlier time points.

Detection of earlier signs of impending regression might be possible by monitoring CD8^+^ T-cell infiltration. Adoptive transfer experiments have shown that control of E.G7-OVA tumour growth depends on retention of CD8^+^ T cells within the tumour. Tumour growth continued when the cells migrated out of the tumour into the spleen and draining lymph nodes (Shrikant and Mescher, 1999). Magnetic resonance imaging detection of immune cell infiltration of a tissue can be accomplished by labelling the immune cells with MR-detectable paramagnetic agents. Using this technique, immune cell infiltration has been detected in the pancreas of an animal model of Type I diabetes (Moore *et al*, 2002). Whether similar experiments will be feasible in a clinical context remains to be determined.

In conclusion, we have demonstrated a noninvasive MRI method that, by detecting the vascular proliferation that precedes immune rejection, can be used to detect the early signs of this process. We propose that DCE-MRI could be used in the clinic to evaluate early tumour responses to immunotherapy. This will, however, require its evaluation in other preclinical models of tumour immune rejection in order to establish the generality of this vascular response. Consideration will also have to be given to selection of the most appropriate pharmacokinetic model used to derive the vascular parameters. An additional complication arises from the fact that tumour perfusion is often grossly heterogeneous and can vary considerably between different tumour types. Therefore, in the clinic DCE-MRI measurements would need to be carried out immediately before and in the days following administration of immunotherapy. While this might require several imaging sessions, this is nevertheless more practical than trying to measure reduction in tumour volume and, more importantly, may give an earlier indication of response.
